# Efficacy of acupuncture on glucocorticoid-induced insomnia: study protocol for a randomized controlled trial assessed by polysomnographic parameters and 5-HT

**DOI:** 10.3389/fneur.2026.1878233

**Published:** 2026-08-18

**Authors:** Yaoxin Chen, Sangyi Lin, Wenlin Xu, Gongyue Zhou, Pei Shen, Fu Xu, Na Zhao, Cong Wang

**Affiliations:** 1 Department of Acupuncture and Moxibustion, The First Affiliated Hospital of Zhejiang Chinese Medical University (Zhejiang Provincial Hospital of Chinese Medicine), Hangzhou, China; 2 Department of Rehabilitation, The First Affiliated Hospital of Zhejiang Chinese Medical University (Zhejiang Provincial Hospital of Chinese Medicine), Hangzhou, China; 3 Department of Massage, The First Affiliated Hospital of Zhejiang Chinese Medical University, (Zhejiang Provincial Hospital of Chinese Medicine), Hangzhou, China; 4 Department of Acupuncture and Moxibustion, Yueyang Hospital of Integrated Traditional Chinese and Western Medicine, Shanghai University of Traditional Chinese Medicine, Shanghai, China

**Keywords:** 5-HT, actigraphy, acupuncture, glucocorticoids, insomnia, polysomnography

## Abstract

**Background:**

Glucocorticoids are extensively utilized in clinical medicine, yet their therapeutic benefits are often accompanied by adverse effects, among which insomnia is particularly prevalent. This sleep disturbance not only compromises treatment efficacy but also significantly diminishes patients’ quality of life. Notably, no targeted therapeutic interventions are available that specifically address glucocorticoid-induced insomnia. Acupuncture therapy has potential as a promising complementary approach for managing sleep disorders in patients receiving glucocorticoid treatment.

**Methods:**

This study presents a randomized, sham-controlled clinical trial protocol. A total of 76 patients with glucocorticoid-induced insomnia will be randomly assigned to either the acupuncture group or the sham acupuncture group. The primary outcome is the change in Pittsburgh Sleep Quality Index total score from baseline to post-intervention. The key secondary outcomes are the changes from baseline to post-intervention in the Insomnia Severity Index score, polysomnography-derived total sleep time, and polysomnography-derived sleep efficiency. Other exploratory outcomes include additional polysomnographic parameters, actigraphy parameters, peripheral 5-hydroxytryptamine levels, emotional status, and daytime functioning.

**Discussion:**

This trial will evaluate the efficacy and safety of acupuncture for glucocorticoid-induced insomnia and explore potential physiological mechanisms in this specific patient population.

**Clinical trial registration:**

https://itmctr.ccebtcm.org.cn/mgt/search, identifier: ITMCTR2025000220.

## 1 Introduction

Glucocorticoids (GCs) were first applied clinically by Hench et al. in 1950 for rheumatoid arthritis treatment, earning the Nobel Prize in Physiology or Medicine the following year (1). Despite their widespread clinical use for anti-inflammatory, immunosuppressive, and antipyretic effects, GCs exhibit numerous adverse effects (2, 3), including hypertension, diabetes mellitus, obesity, osteoporosis, and peptic ulcer exacerbation (potentially leading to hemorrhage or perforation) (4), and even cause neurological symptoms. Neuropsychiatric complications may manifest as personality changes, psychosis, or even epileptic seizures (5).

Among these, glucocorticoid-induced insomnia (GII)—characterized by excitation, agitation, anxiety, depression, or mania—represents the most prevalent complication (6). The clinical manifestations of GII include sleep initiation/maintenance difficulties, reduced sleep quality, and decreased total sleep time (TST), warranting clinical attention, as chronic insomnia may exacerbate pre-existing psychiatric symptoms and compromise GCs therapeutic outcomes (7).

To our knowledge, no randomized controlled trial has specifically targeted glucocorticoid-induced insomnia, and current management generally follows established protocols for primary insomnia. Conventional treatments for insomnia include pharmacotherapy (benzodiazepines, nonbenzodiazepine hypnotics, antidepressants, and antihistamines) and nonpharmacological interventions. While rapid-acting, pharmacological options carry risks of hangover effects, cognitive impairment, and dependence (8). Cognitive behavioral therapy for insomnia (CBT-I) and other psychotherapeutic approaches are generally accepted internationally as the primary recommended treatment for chronic insomnia disorder (9). However, due to their time-intensive nature and because physicians need much professional training to conduct them, these methods are not fully implemented in China. Other guideline-endorsed modalities (light therapy, physical therapy, and sleep hygiene education) demonstrate limited efficacy and stability, with the European Insomnia Guidelines rating evidence for light therapy and physical therapy as low quality (10).

Traditional Chinese Medicine (TCM) is effective for treating insomnia (11, 12), yet the literature reveals no clinical studies on TCM approaches for treating GII. Acupuncture is a branch of TCM and offers distinct advantages as a simple, accessible, and cost-effective intervention, with demonstrated improvements in sleep efficiency (SE), daytime functioning, mood regulation, and sleep architecture (13–15). Our prior randomized controlled trial (RCT) revealed that acupuncture has better clinical efficacy and safety for insomnia than sham acupuncture. It significantly improved sleep quality and daytime function, reduced insomnia severity, and adjusted mood in insomnia patients (16). Acupuncture for insomnia has a profound theoretical basis as well as an evidence base. However, the effects of acupuncture on GII remain unexplored. Therefore, this study is a RCT designed to explore the effect of acupuncture on the treatment of GII. It introduces a multidimensional evaluation scheme to comprehensively assess the specific efficacy and safety of acupuncture for GII.

In addition to the conventional subjective sleep assessment scale, polysomnography (PSG) and actigraphy (ACT) will be used in this study. The strategy of using PSG for screening enrolled patients and both PSG and ACT for monitoring sleep changes pre−/post-intervention offers distinct advantages. PSG remains the gold standard for detailed sleep architecture analysis, providing high-resolution data on sleep stages (e.g., rapid eye movement sleep, nonrapid eye movement sleep), respiratory events, and limb movements through multimodal recordings (17, 18). However, its laboratory setting and intrusive nature may alter natural sleep patterns. To solve this problem, we will use PSG data from the second night for analysis to reduce the “first-night effect” and will use ACT as a complementary indicator of objective sleep. ACT, although less precise in sleep staging, enables long-term, unobtrusive monitoring in real-world environments, capturing sleep–wake patterns over extended periods (19). Circadian rhythm disruption is likely one of the core mechanisms underlying GII (20). In addition, dysregulation of the hypothalamic–pituitary–adrenal (HPA) axis serves as a pivotal pathological mechanism in sleep disturbances. The rationale for monitoring serotonin 5-HT levels lies in its critical role in insomnia for the HPA axis. GCs disrupt the HPA axis and interact with serotonergic neurotransmission (21), which is pivotal in sleep–wake regulation (22). Modern mechanistic research suggests that GCs can cause hyperpolarization of nerve cell membranes, selectively inhibit spontaneous electrical activity, and enhance the activity of dopamine *β*-hydroxylase and phenylethanolamine N-transmethylase, increase the synthesis of norepinephrine and epinephrine, inhibit the activity of tryptophan hydroxylase, reduce the concentration of central 5-HT, and lead to mental abnormalities. This is an important mechanism for causing insomnia (23, 24). Although peripheral 5-HT is predominantly synthesized outside the central nervous system (e.g., in the gut and platelets), some evidence suggests that its levels may be influenced by central serotonergic activity via indirect pathways such as the vagus nerve and inflammatory signals (25). This study will include peripheral serum 5-HT as an exploratory biomarker to generate preliminary mechanistic insights into acupuncture treatment for GII.

Based on our literature search, no prior randomized controlled trial has specifically investigated the therapeutic efficacy of acupuncture for glucocorticoid-induced insomnia while incorporating comprehensive biomarker analysis. The present study employs a rigorous design to address this gap. Based on this framework, we proposed two testable hypotheses: (1) Acupuncture significantly improves both subjective sleep scales and objective sleep parameters compared with those of sham acupuncture in GII patients. (2) The clinical benefits of acupuncture correlate with measurable changes in peripheral 5-HT levels, suggesting that the modulation of serotonergic pathways is a potential mechanism for the therapeutic effects of acupuncture in GII patients.

## 2 Methods/design

### 2.1 Study design

**Figure 1:**
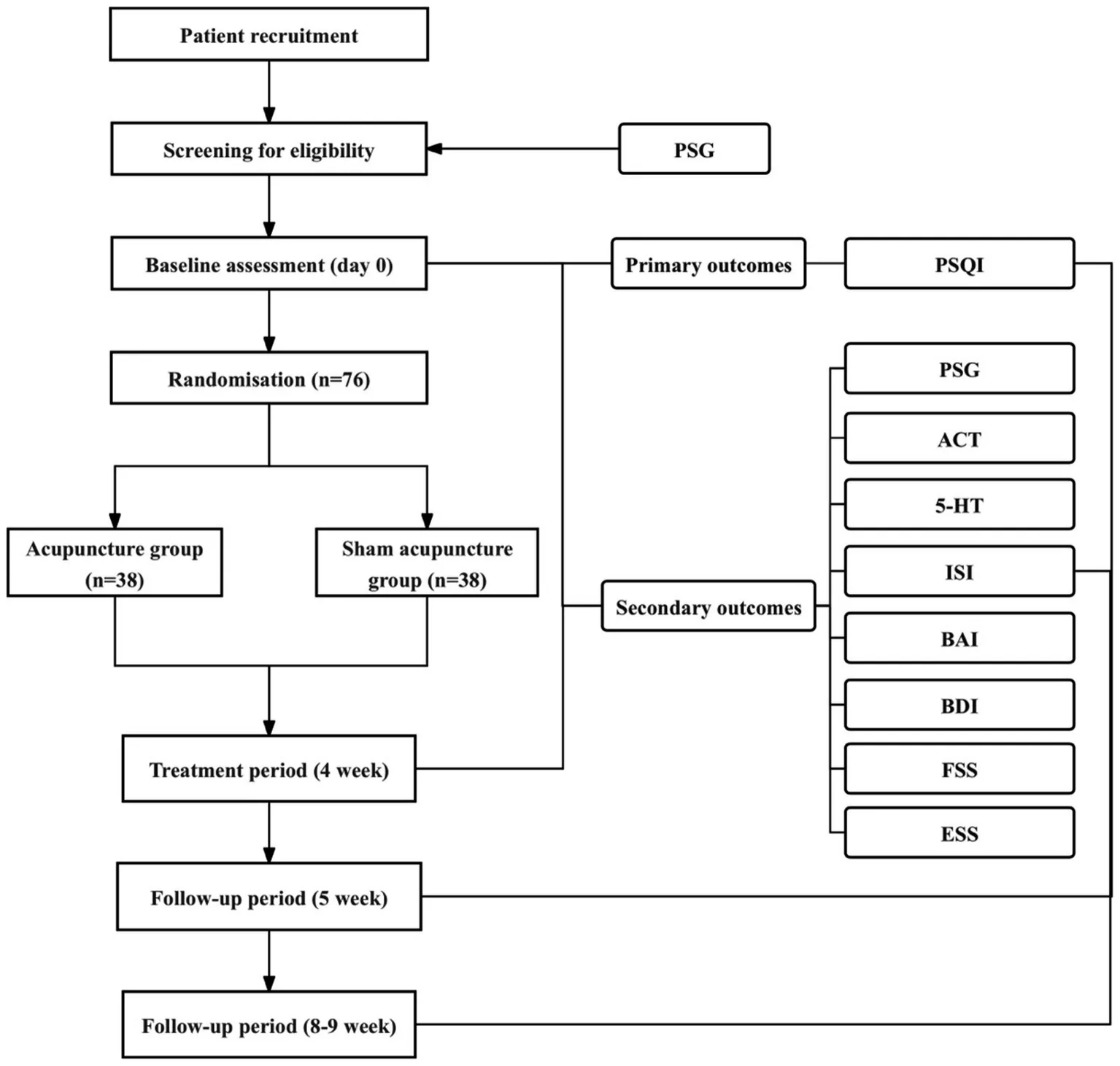
Flow diagram. BAI, Beck Anxiety Inventory; BDI, Beck Depression Inventory; FSS, Fatigue Severity Scale; ESS, Epworth Sleepiness Scale; ISI, Insomnia Severity Index; PSG, Polysomnography; PSQI, Pittsburgh Sleep Quality Index; 5-HT, 5-Hydroxytryptamine.

This study is designed as a single-centre, sham-controlled, randomized controlled trial. This clinical trial will be conducted at the Acupuncture Department of the First Affiliated Hospital of Zhejiang Chinese Medical University. This trial was rigorously designed according to the Consolidated Standards of Reporting Trials (CONSORT) guidelines and the Standards for Reporting Interventions in Clinical Trials of Acupuncture (STRICTA) (Table 1). In addition, the study protocol strictly adheres to the Standard Protocol Items: Recommendations for Interventional Trials (SPIRIT) guidelines (See the supplementary material). The study flowchart and procedure are depicted in Figure 1 and Table 2.

**Table 1:** STRICTA of acupuncture treatment.

Item	Detail	Description
1. Acupuncture rationale	(1a) Style of acupuncture	Traditional Chinese Medicine
	(1b) Reasoning for treatment provided, based on historical context, literature sources, and/or consensus methods, with reference where appropriate	Textbook of the meridian, literature review of acupuncture for insomnia
	(1c) Extent to which treatment was varied	Formularized
2. Details of needling	(2a) Number of needle insertions per subject per session	4
	(2b) Names	HT7, KI7
	(2c) Depth of insertion, based on a specified unit of measurement, or on a particular tissue level	HT7: Vertical insertion, 0.3–0.5 cun; KI7: Vertical insertion, 0.5–1.0 cun
	(2d) Response sought	de qi
	(2e) Needle stimulation	manual
	(2f) Needle retention time	20 min
	(2g) Needle type	0.25 × 40 mm needles (Andi, Guizhou, China), stainless steel
3. Treatment regimen	(3a) Number of treatment sessions	10
	(3b) Frequency and duration of treatment sessions	Three sessions per week for the first 3 weeks and one session in the final (fourth) week, giving a total of 10 sessions.
4. Other components of treatment	(4a) Details of other interventions administered to the acupuncture group	none
	(4b) Setting and context of treatment, including instructions to practitioners, and information and explanations to patients	All interventions will be administered at a same clinical site by two licensed acupuncturists who will receive identical standardized training in the study protocol prior to trial initiation.
5. Practitioner background	(5) Description of participating acupuncturists (qualification or professional affiliation, years in acupuncture practice, other relevant experience)	Two licensed acupuncturists (minimum 3 years clinical experience).
6. Control or comparator interventions	(6a) Rationale for the control or comparator in the context of the research question, with sources that justify this choice	This method optimizes blinding integrity and participant expectancy while providing a credible placebo comparator with minimal physiological activity.
	(6b) Precise description of the control or comparator. If sham acupuncture or any other type of acupuncture-like control is used, provide details as for Items 1 to 3 above.	This study employs the Streitberger needle as the control intervention. See details in methods.

**Table 2:** Trial processes chart.

Week	0	1	2	3	4	5, 8–9
	Baseline	Treatment phase				Follow-up phase
PATIENTS						
Telephone reservation	**×**					
Enrolment	**×**					
Sign informed consent	**×**					
Clinical interview	**×**					
Sleep scales	**×**					
Physical examination	**×**				**×**	
Laboratory test	**×**				**×**	
PSG	**×**				**×**	
GROUPS						
Acupuncture group		Ten treatments				
Sham- acupuncture group		Ten treatments				
OUTCOME MEASUREMENT						
PSQI	**×**				**×**	**×**
ISI	**×**				**×**	**×**
PSG	**×**				**×**	
ACT	**×**				**×**	
5-HT	**×**				**×**	
BAI	**×**				**×**	
BDI	**×**				**×**	
FSS	**×**				**×**	
ESS	**×**				**×**	
Sleep diary	**×**	**×**	**×**	**×**	**×**	
Success of subject-blinding test					**×**	
ADVERSE EVENTS						
Reasons for dropouts or withdrawals		**×**	**×**	**×**	**×**	**×**
Patient’s compliance					**×**	**×**

ACT, Actigraphy; BAI, Beck Anxiety Inventory; BDI, Beck Depression Inventory; ESS, Epworth Sleepiness Scale; FSS, Fatigue Severity Scale; ISI, Insomnia Severity Index; PSG, Polysomnography; PSQI, Pittsburgh Sleep Quality Index; 5-HT, 5-hydroxytryptamine.

### 2.2 Participants and setting

We will recruit eligible participants who meet the operational definition of glucocorticoid-induced insomnia described below from the Acupuncture Department of the First Affiliated Hospital of Zhejiang Chinese Medical University. Subjects will be randomly assigned to 2 parallel groups based on randomization numbers: the acupuncture group and the sham acupuncture group, with an enrolment ratio of 1:1. Written informed consent will be obtained from all participants. This study was approved by the ethics committee of the First Affiliated Hospital of Zhejiang Chinese Medical University (no. 2024-KLS-089-02). This study will be conducted in accordance with the Declaration of Helsinki.

Operational definition of glucocorticoid-induced insomnia: In this trial, GII is operationally defined as insomnia meeting the applicable ICSD-3 diagnostic criteria (26) after documented systemic glucocorticoid exposure. Eligible patients must have either new-onset insomnia or a clear worsening of pre-existing mild sleep complaints that did not meet full ICSD-3 criteria before the index exposure, with full diagnostic criteria first met within 14 days after glucocorticoid initiation or clinically meaningful dose escalation, while receiving glucocorticoids at ≥5 mg/day prednisone equivalent. Patients who met full ICSD-3 criteria for insomnia before the index glucocorticoid exposure will not be eligible. The diagnosis will be based on a structured clinical interview, medical record review, a 1-week sleep diary, PSQI >5, ISI assessment, and baseline polysomnography showing objective total sleep time <6 h, consistent with recommended insomnia assessment procedures (27).

Glucocorticoid exposure will be recorded in detail, including drug type, route, daily prednisone-equivalent dose, administration time, start date, dose-escalation date, treatment duration, and dose stability. This information is collected because glucocorticoid-associated sleep disturbance has been reported in clinical and mechanistic studies and may be influenced by glucocorticoid dose, duration, and circadian timing (24, 28). Patients who met full ICSD-3 criteria for insomnia before the index glucocorticoid initiation or dose escalation, those with prior regular sedative-hypnotic use, and those with other primary sleep disorders will be excluded. Potential confounders, including underlying disease activity, pain, emotional status, caffeine or alcohol use, and concomitant sleep-affecting medications, will be documented at baseline and follow-up. Unauthorized sedative-hypnotic use and major changes in glucocorticoid exposure or other sleep-affecting medications during the trial will be recorded. A major change in glucocorticoid exposure is predefined as either (1) a > 50% change in daily prednisone-equivalent dose relative to baseline or (2) initiation or cessation of systemic glucocorticoid therapy during the treatment phase. These events will be addressed in per-protocol and sensitivity analyses.

The study will span 8–9 weeks, comprising a screening phase, a treatment phase and a follow-up phase (1-week follow-up and 1-month follow-up). The 1-week follow-up was selected to capture the most direct and immediate effects following intervention completion, as 1 week is sufficient for participants to adopt and adapt to new sleep patterns. Compared with more frequent assessments (e.g., every few days), the 1-week interval imposes less burden on participants, thereby enhancing study retention and data quality. The 1-month follow-up was chosen because it represents a critical time point for evaluating whether therapeutic benefits are sustained after the intervention is withdrawn. From a clinical perspective, poor sleep persisting for more than 1 month is generally considered one of the important time thresholds for diagnosing insomnia (29); therefore, the 1-month assessment helps determine whether the intervention has truly modified the disease trajectory rather than merely providing transient relief.

#### 2.2.1 Inclusion criteria

(1) Met the above diagnostic criteria for insomnia according to the ICSD-3 criteria;
(2) Age 18–65 years, gender is not limited;
(3) A total PSQI score >5;
(4) Objective TST of <6 h through PSG;
(5) Participants receiving systemic glucocorticoid therapy at a dose of ≥5 mg/day prednisone equivalent, with either new-onset insomnia or a clear worsening of pre-existing mild sleep complaints that did not meet full ICSD-3 criteria before the index exposure, and with full diagnostic criteria first met within 14 days after glucocorticoid initiation or dose escalation. Glucocorticoid type, route, daily prednisone-equivalent dose, administration time, start date or dose-escalation date, and planned dose adjustment will be recorded.
(6) No sedative-hypnotic medications were taken prior to participation in this study;
(7) No acupuncture interventions were administered during the 6-month period prior to the study;
(8) Voluntarily provided written informed consent.

#### 2.2.2 Exclusion criteria

(1) Those with severe cardiac, hepatic or renal functional impairment and respiratory, haematologic or psychiatric system diseases;
(2) Patients who met full ICSD-3 diagnostic criteria for insomnia before the index glucocorticoid initiation or dose escalation; mild pre-existing sleep complaints that did not meet full ICSD-3 criteria will not, by themselves, constitute an exclusion criterion;
(3) Patients with unstable underlying disease, severe pain, acute infection, or other clinical conditions judged by investigators to be the primary cause of insomnia;
(4) Patients who used sedative-hypnotics before participation, or who initiated or substantially changed other medications known to affect sleep within 2 weeks before screening, including antidepressants, antipsychotics, opioids, stimulants, or other centrally acting medications, unless the regimen for those other medications is stable and can be documented;
(5) Lactating or pregnant women;
(6) Patients with infectious diseases, such as those suffering from tuberculosis, hepatitis or acquired immune deficiency syndrome;
(7) People who are not suitable for acupuncture treatment, such as those with unhealed trauma, severe skin diseases, allergies;
(8) Sleep PSG showing a sleep apnoea-hypopnea index ≥10/h;
(9) Sleep PSG showing periodic limb movements during sleep>15 times/h;

#### 2.2.3 Withdrawal criteria

(1) Patients who were lost to contact during the treatment or follow-up phase;
(2) Patients who refused to continue treatment during the treatment phase;
(3) Those who discontinued treatment due to adverse events, which were recorded;
(4) Patients whose disease progressed during the course of the study, making them unsuitable for continuation;
(5) Patients who did not comply with the subject research process after entering the study;
(6) Those who were difficult to assess due to poor adherence to and noncompliance with medical advice during the course of treatment;
(7) Those who take sedative-hypnotic drugs without authorization in the course of treatment.

#### 2.2.4 Sample size

The sample size was determined using the following formula for comparing means between two groups from Medical Statistics (edited by Sun Zhenqiu) (30):

n=2[(Zα/2+Zβ)δσ]2

Based on previous published findings (31), sham acupuncture resulted in a post-treatment PSQI score reduction to 11.69 ± 3.35 (mean±SD), with a sample standard deviation (s) of 3.35. We hypothesized that acupuncture would yield an additional 2.70-point reduction in PSQI scores compared with that associated with sham acupuncture. The statistical hypothesis test of the evaluation variable is two-sided, and the significance level is set at 5%. The type 2 error (*β*) will be set to 0.1. The calculation yielded n = 31.52 ≈ 32 per group. Accounting for a 20% dropout rate based on clinical experience, the final required sample size was 38 participants per group (76 total participants).

#### 2.2.5 Randomization

An independent statistician generated a 1:1 allocation sequence in SPSS 25.0 (IBM Corp.) using permuted blocks of undisclosed varying sizes. The master allocation list and random seed are retained by the statistician in a restricted-access file until database lock.

#### 2.2.6 Blinding

The allocation sequence is implemented using sequentially numbered, opaque, sealed envelopes prepared by personnel not involved in participant enrolment. Each envelope contains the treatment card corresponding to the next allocation in the sequence. After eligibility is confirmed and written informed consent is obtained, the treating acupuncturist opens the next envelope in numerical order immediately before the first treatment. Envelope integrity and the date and time of opening are documented.

Emergency envelopes labeled with “Emergency Unblinding Document: Clinical Trial of Acupuncture for GII,” containing group assignments (e.g., “Participant ID XXX: [Group]”), were prepared identically to treatment envelopes. Unblinding was permitted only for (1) serious adverse events or (2) life-threatening emergencies requiring immediate intervention, following approval by the principal investigator. Participants with unplanned unblinding will be excluded from the PPS but retained in the FAS and safety analyses.

The database will be locked after blind review and resolution of all data queries. Group allocation will be revealed to the principal investigator and statistician only for the final analysis. Blinding will be considered compromised if full allocation disclosure occurs or if >20% of emergency envelopes are opened during the trial.

This is a single-blind trial with participants blinded to treatment allocation. The acupuncturists are not blinded due to the nature of the intervention. However, all outcome assessors (including those administering the PSQI, ISI, and other questionnaires), PSG scorers, actigraphy analysts, laboratory personnel performing the 5-HT assays, and statisticians are blinded to group assignment until database lock. To assess participant blinding success, a standardized questionnaire will be administered upon completion of the intervention, asking participants to guess their allocated group and rate their confidence. Bang’s Blinding Index will be calculated to quantify the adequacy of blinding. Emergency unblinding is permitted only under predefined conditions (SAEs or life-threatening emergencies) and requires principal investigator approval. Participants with unplanned unblinding will be retained in the FAS and safety analyses but excluded from the PPS as a major protocol violation.

### 2.3 Intervention

The acupuncturists for this study received a master’s degree in acupuncture and tuina at Zhejiang Chinese Medical University, have 8 years of training experience, obtained a doctor qualification certificate and have 3 years of clinical work experience. Participants in both the acupuncture and sham control groups will receive interventions three sessions per week for the first 3 weeks and one session in the final (fourth) week, for a total of 10 sessions.

#### 2.3.1 Acupuncture group

**Figure 2:**
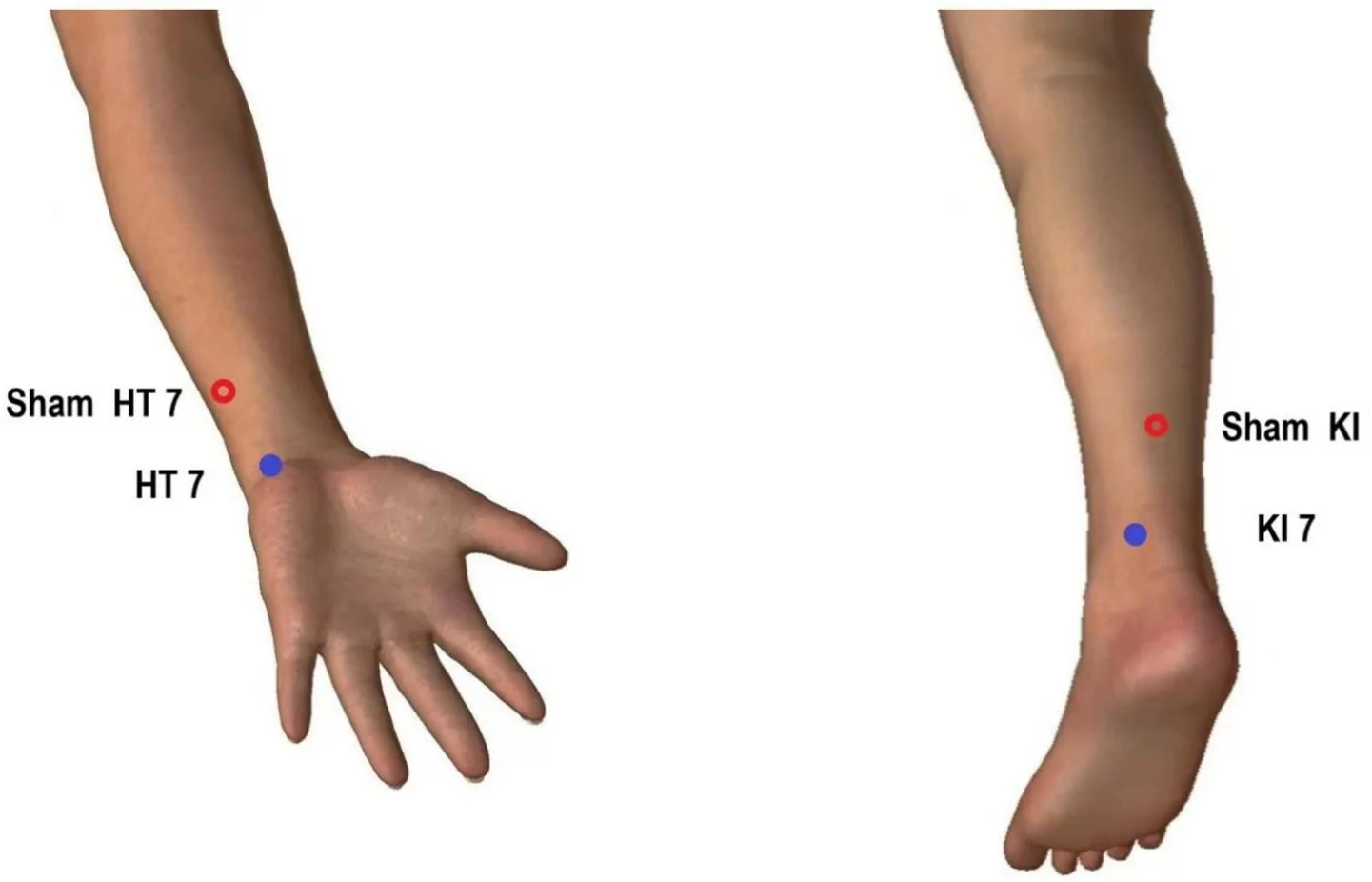
Acupoints for the acupuncture and sham control acupuncture groups.

The subjects in the acupuncture group will be placed in the supine position. We selected two acupoints for this study: HT7 and KI7. The reason for choosing these acupoints is based on our previous clinical study, which was based on the theoretical framework of TCM according to the Heart-Kidney interaction theory (16). Participants will assume the supine position after the skin is disinfected with 75% alcohol. A disposable sterile stainless steel acupuncture needle with a length of 40 mm and a diameter of 0.25 mm will be used (Andy, Guizhou, China). Acupoint localization will follow GB/T12346-2021 national standards (Table 3; Figure 2): bilateral HT7: 0.3–0.5 cun ( ≈ 6–10 mm) perpendicular insertion, avoiding ulnar vasculature; bilateral KI7: 0.5–1.0 cun ( ≈ 10-20 mm) perpendicular insertion. After piercing, thrusting and twirling of the needle will be performed to induce the sensation of ‘De qi’, and the needle will be left for 20 min (32). De qi means that after the needle has penetrated into the acupoint to a certain depth, the needle is thrusted and twirled to cause the acupoint to obtain the induction of meridian qi. The subjects will have a self-conscious reaction, such as soreness, heaviness or distention. This is the key process in acupuncture treatment (33, 34).

**Table 3:** Locations of acupuncture points.

Acupoint	Location
HT7	At the ulnar end of the transverse crease of the wrist, in the depression on the radial side of the tendon of muscle flexor carpi ulnaris
KI7	2.0 cun ( ≈40mm ) above the medial malleolus (inner ankle bone), anterior to the Achilles tendon, in the depression between the tibia (shinbone) and the Achilles tendon
Sham HT7	Located about 2.0 cun away from the HT7 along the direction of the ulnar flexor carpi tendon, avoiding the ulnar flexor carpi tendon, at the junction of the red and white flesh between the Heart Meridian and Small Intestine Meridian.
Sham KI7	Located 2.0 cun lateral to the inner belly of the gastrocnemius muscle from KI7, avoiding the course of the Kidney Meridian.

#### 2.3.2 Sham control group

Subjects who are assigned to the sham control group will receive placebo acupuncture treatment with a noninvasive placebo needle of sham acupoints (35). The Streitberger needle’s device will be positioned on the skin surface at the target sham acupoints, with its external appearance closely resembling that of genuine acupuncture needles. Upon gentle tapping by the practitioner, the blunt tip will retract into the handle assembly, creating a convincing illusion of needle insertion. This mechanism produces both visual and tactile simulations of skin penetration through apparent shortening of the needle shaft while maintaining complete epidermal integrity through a nonpenetrating design. The Streitberger needle has been widely used in clinical research on placebo acupuncture (36–38), and studies have shown that using this needle in a placebo acupuncture group is reliable for the Chinese population (39). Sham HT 7 is 2 cuns (≈40 mm) lateral to HT 7, and sham KI 7 is 2 cuns (≈40 mm) lateral to KI 7 (Table 3; Figure 2). The purpose of choosing the sham points in this way has been explained in previous similar clinical studies (40); this approach avoids the true acupoints and meridian routes. Choosing sham points close to the true acupoints was conducive to blinding.

### 2.4 Outcomes

#### 2.4.1 Primary efficacy outcome

The primary efficacy outcome is the change in the Pittsburgh Sleep Quality Index (PSQI) total score from baseline to post-intervention.

#### 2.4.2 Secondary efficacy outcomes

##### 2.4.2.1 Subjective efficacy evaluation of insomnia

Changes in Insomnia Severity Index (ISI) scores before and after treatment, both within and between groups, served as subjective efficacy indicators for insomnia.

##### 2.4.2.2 Objective efficacy evaluation of insomnia

(1) Objective sleep parameters, measured via polysomnography (NIHON KOHDEN, Japan) on the second night, included TST; sleep onset latency; rapid eye movement sleep (REMS) latency; wake after sleep onset; frequency of awakenings; sleep efficiency; number of microarousals; arousal index; and percentages of N1, N2, N3, and REM sleep (N1%, N2%, N3%, R%). Only data from the second night of the monitoring phase were analyzed for efficacy assessment. PSG is resource-intensive and burdensome, requiring overnight laboratory stays, and repeated assessments at multiple follow-up points would increase dropout risk and costs, while possibly introducing practice effects or sleep disturbances from the unfamiliar environment. In contrast, subjective questionnaires (PSQI, ISI) are non-invasive, easily administered remotely, and suitable for repeated measurements. Therefore, PSG was prioritized for pre- and post-treatment assessments to verify immediate physiological effects, whereas subjective scales were used to track longer-term clinical outcomes.
(2) Actigraphy (ActiLife, United States), which uses a motion sensor worn on the nondominant wrist to assess body movements, serves as an objective indicator for evaluating sleep–wake states. The changes in the number of awakenings, total wake time (TWT), total sleep time (TST), and sleep efficiency before and after treatment will be measured using actigraphy (41).
(3) Fasting venous blood will be collected pre−/post-intervention (08,00–09:00) for serum 5-HT analysis. Changes in these parameters before and after acupuncture intervention will be used to assess objective therapeutic efficacy.

##### 2.4.2.3 Evaluation of emotional state and daytime functioning

Changes in the Beck Anxiety Inventory (BAI) and Beck Depression Inventory (BDI) scores before and after treatment will be used to evaluate emotional state, whereas changes in the Fatigue Severity Scale (FSS) and Epworth Sleepiness Scale (ESS) scores will be used to assess daytime functioning.

To address multiplicity while maintaining a clear interpretative framework, three outcomes are prespecified as key secondary endpoints: (1) change in PSG-derived TST from baseline to post-treatment; (2) change in PSG-derived sleep efficiency from baseline to post-treatment; and (3) change in ISI score from baseline to post-treatment. These three endpoints will be subject to formal false discovery rate control. All remaining secondary outcomes are exploratory and will be presented with nominal *p*-values without multiplicity adjustment for hypothesis-generating purposes.

### 2.5 Safety evaluation

#### 2.5.1 Acupuncture safety

Safety will be evaluated by comparing pre- and post-treatment laboratory results, including complete blood count, liver function tests (ALT, AST), and renal function tests (BUN, Cr).

#### 2.5.2 Adverse events

AEs will be recorded and include needle-related reactions such as fainting during acupuncture (needle syncope), a stuck needle (needle retention), subcutaneous bleeding, haematoma, and other unexpected events. AEs will be classified into three severity levels: mild (no impact on normal functioning), moderate (partial impact on normal functioning), and severe (significant impairment of normal functioning). The causality of AEs will be assessed using a four-level classification: definitely related, probably related, possibly unrelated, and unrelated/undetermined. Additionally, a serious adverse event (SAEs) form will be used to document major safety incidents during the trial. In the context of acupuncture, SAEs encompass traumatic pneumothorax, subarachnoid hemorrhage, injuries to the brain, spinal cord, heart, blood vessels, liver, spleen, kidneys, and gallbladder; hepatitis B, hepatitis C, AIDS, syphilis; other severe local or systemic infections; severe neurologic or vascular injuries; permanent disability; and death (42). SAEs will be reported to the principal investigator within 24 h and to the institutional ethics committee within 72 h, with follow-up until resolution or stabilization.

### 2.6 Statistical analyses

Statistical analyses will employ both the full analysis set (FAS) and per protocol set (PPS) approaches.

#### 2.6.1 Data sets definition

FAS: All randomized participants who received at least one intervention and had at least one post-baseline efficacy assessment. This set will be considered a modified intention-to-treat (ITT) principle. Participants who experience major changes in glucocorticoid exposure will be retained in the FAS.

PPS: Subjects who met inclusion/exclusion criteria, completed ≥80% of acupuncture sessions, and had no major protocol violations (e.g., unauthorized use of sedative-hypnotic medications). Participants who experience major changes in glucocorticoid exposure will be excluded from the per-protocol set.

Safety Set (SS): All subjects who received at least one intervention and had safety data recorded. Participants who experience major changes in glucocorticoid exposure will be retained in the SS.

Cases with unauthorized use of sedative-hypnotic medications will be excluded from the PPS but retained in the FAS and SS analyses.

#### 2.6.2 Primary and secondary analyses

All statistical tests will be two-sided, with *p* < 0.05 considered statistically significant for the primary outcome. The primary analysis will use a mixed model for repeated measures with PSQI total scores at all post-baseline visits (post-treatment, 1-week follow-up, and 1-month follow-up) as the dependent outcomes. Group, visit, and the group-by-visit interaction will be included as fixed effects, and baseline PSQI score will be entered solely as a covariate. Within-participant correlations will be modeled using an unstructured covariance matrix, and parameters will be estimated by restricted maximum likelihood. The adjusted between-group mean difference at the post-treatment visit, with its 95% confidence interval, is prespecified as the primary comparison; the 1-week and 1-month contrasts are secondary longitudinal comparisons.

For continuous secondary outcomes measured repeatedly, MMRM or generalized estimating equations will be used as appropriate to the outcome distribution and measurement schedule. The primary MMRM will use all available post-baseline observations under a missing-at-random assumption without single imputation. Multiple imputation will be used as a supportive sensitivity analysis, and a delta-adjusted pattern-mixture analysis will assess robustness to departures from the missing-at-random assumption. Major changes in glucocorticoid exposure will be treated as intercurrent events: participants will remain in the FAS and SS and their observed outcomes will be included in the primary treatment-policy analysis, whereas they will be excluded from the PPS. Glucocorticoid dose stability (stable vs. unstable according to the predefined criteria) will be included as a binary covariate in a prespecified sensitivity analysis, rather than in the primary model.

For the three key secondary endpoints (change from baseline to post-treatment in ISI score, PSG-derived sleep efficiency, and PSG-derived TST), the Benjamini-Hochberg procedure will control the false discovery rate at 5%. The remaining exploratory outcomes will be reported with nominal *p*-values without adjustment and interpreted as hypothesis-generating.

#### 2.6.3 Descriptive statistics

Continuous variables following a normal distribution will be expressed as the mean±standard deviation. Non-normally distributed data will be expressed as the median (minimum, maximum) [M, (min, max)]. Categorical data will be expressed as frequencies, rates, or proportions. Between-group comparisons of categorical variables will be conducted using the χ2 test or Fisher’s exact test. Rank-sum tests will be used for ordinal variables.

### 2.7 Quality control and data monitoring

Study data will be collected at four time points: baseline (1 week before the initial intervention), post-treatment (on the final treatment day), 1-week follow-up (days 6–8 post-treatment), and 1-month follow-up (days 26–36 post-treatment). Data collection will comprise three components: (1) Participants will independently complete standardized assessments at the research centre under supervision, with a dedicated assessment physician available for consultation during the approximately 30-min evaluation period; (2) participants will maintain home sleep diaries for 1 week during each assessment phase under physician guidance; and (3) eligible participants will undergo two consecutive nights of inpatient PSG monitoring before and after the intervention period. At baseline and each follow-up visit, investigators will record the indication for glucocorticoid therapy, underlying disease status, glucocorticoid type, route, dose, timing of administration, treatment duration, dose changes, and concomitant medications with potential sleep effects. Pain intensity, emotional status, caffeine/alcohol use, and sleep diary information will also be collected to assess potential confounding factors.

Dedicated research personnel will receive specialized training in data entry, coding, and secure storage protocols prior to handling study data. Similarly, data analysts will complete comprehensive training in data evaluation and statistical analysis methods prior to performing study analyses.

## 3 Discussion

This randomized controlled trial will evaluate the clinical efficacy and safety of acupuncture for GII and explore potential mechanisms. The multidimensional assessment framework will combine PSG, actigraphy, and peripheral 5-HT measurements to characterize subjective, objective, and exploratory biomarker outcomes. The study is expected to provide preliminary evidence regarding, rather than presuppose, the role of acupuncture in GII management.

### 3.1 Pathophysiological and theoretical framework

The primary mechanism by which GCs cause insomnia is via negative feedback disorders of the HPA axis. Under physiological conditions, GCs are secreted through the hypothalamus-pituitary–adrenal axis (HPA axis) and follow a circadian rhythm (peak in the early morning and trough at night). Exogenous GCs can cause a continuous increase in GCs, which inhibits the negative feedback of the HPA axis and delays the “quiescent period” of cortisol secretion, which manifests as an abnormal increase in GCs levels at night (43–45). As observed in anxiety-related sleep disturbances, HPA hyperactivity is correlated with sleep maintenance difficulties (46). In addition, GCs can activate the prefrontal cortex and limbic system, specifically by increasing glutamate release in the prefrontal cortex through membrane-associated mineralocorticoid receptors, reducing GABAergic inhibition, and increasing neuronal excitability, resulting in increased wakefulness and insomnia (47). In addition, sustained high GCs lead to atrophy of the dendrites of prefrontal pyramidal cells and lengthening of excitatory synapses in the basolateral nucleus of the amygdala, triggering anxious wakefulness and thus causing insomnia (48, 49). Finally, GCs can also overactivate glucocorticoid receptors, inhibit hippocampal neurogenesis and synaptic plasticity, impair sleep-dependent memory consolidation, and indirectly affect sleep quality (50, 51).

GII is closely linked to the 5-HT system. Under physiological conditions, central 5-HT promotes non-rapid eye movement sleep (NREMS) by inhibiting wake-promoting neurons (e.g., noradrenergic neurons in the locus coeruleus) and activating GABAergic neurons in the ventrolateral preoptic nucleus of the hypothalamus (52). In the pathological context of GC interference, acute GC exposure can increase 5-HT release; however, because it simultaneously inhibits 5-HT₁A receptors, this may attenuate the sleep-promoting effect. Chronic GC exposure can reduce the expression of 5-HT synthase and decrease 5-HT availability, which may in turn lower the excitability of sleep-promoting VLPO neurons and contribute to insomnia (53). The main sleep manifestations associated with GC-induced insomnia include reduced slow-wave sleep and REM sleep, decreased sleep spindle and delta (slow-wave) power, and increased gamma oscillation (a marker of wakefulness), resulting in a shallow sleep state (47). The underlying mechanisms are thought to involve modulation of HPA axis activity and the serotonergic system.

It is important to note that in this study, peripheral serum 5-HT is used only as an exploratory biomarker. Peripheral 5-HT levels are influenced by multiple factors and should not be interpreted as a direct surrogate of central serotonergic activity. Consequently, all mechanistic inferences drawn from these data should be considered exploratory and hypothesis-generating, rather than definitive conclusions.

### 3.2 Evidence-based acupoint selection

Acupuncture has significant potential as a nonpharmacological intervention for insomnia across diverse patient populations, as supported by multiple studies (54–56). Evidence indicates efficacy, particularly for post-stroke insomnia, with intradermal acupuncture significantly reducing sympathetic hyperactivity (measured by the LF/HF ratio), improving sleep scales (ISI), and outperforming sham procedures (57). Among cancer survivors, especially breast cancer patients, insomnia related to endocrine therapy and anxiety can be addressed with acupuncture (58). For depression-related insomnia, meta-analyses have shown that acupuncture alone significantly reduces both insomnia severity (PSQI) and depressive symptoms (HAMD) compared with that of placebo, demonstrating comparable or superior efficacy to pharmacotherapy for active depression (56). Collectively, the above studies, ranging from clinical trials to systematic reviews, provide robust evidence for integrating acupuncture into insomnia management strategies across various clinical contexts.

According to our group’s previous RCT (16) of applying the HT7 and KI7 points to treat chronic insomnia, compared with the sham acupuncture group, the acupuncture group can decrease PSQI and ISI scores and improve sleep structure by decreasing the sleep latency, decreasing NREM stage 1 sleep and increasing NREM stage 3 sleep (16). A comprehensive meta-analysis by Pan (59) systematically evaluated acupoint utilization patterns in insomnia treatment over the past decade, identifying 68 principal acupoints and 88 supplementary points. This analysis revealed Shenmen as the most frequently employed acupoint (69.16% utilization rate), leading to its classification as a first-line therapeutic point for insomnia management. Clinical studies have consistently demonstrated HT7’s significant therapeutic effects for insomnia (60, 61), with mechanistic research showing its ability to activate key brain regions involved in sleep–wake regulation and emotional control within just 10 min of stimulation (62). Similarly, KI7 has emerged as a particularly effective acupoint (demonstrating >90% response rates) for heart-kidney disharmony subtype insomnia (63, 64). Neuroimaging studies have revealed the capacity of KI7 to modulate widespread neural networks, including the parietal lobe, inferior parietal lobule, supramarginal gyrus, cingulate gyrus, brainstem, cerebellar anterior lobe, thalamus, midbrain, temporal lobe, superior temporal gyrus, and precuneus-regions functionally connected to the foot-shaoyin kidney meridian pathways (65).

According to the theory of TCM, endogenous GCs demonstrate distinct circadian rhythmicity in their secretion patterns, with peak levels occurring between 08:00 and 10:00, followed by a gradual decline to nadir levels at 24:00. Exogenous glucocorticoid administration induces kidney yang hyperactivity, creating yang excess that disrupts yin-yang equilibrium (66). This impairs the normal nocturnal submersion of yang into yin, compromising yin’s tranquilizing function and manifesting as insomnia with yang hyperactivity syndrome. Prolonged yang excess leads to yin depletion, resulting in heart-blood deficiency and shen (spirit) malnourishment. This transition is characterized by persistent insomnia accompanied by fatigue, lethargy, and impaired concentration. This dual-phase pathogenesis implicates both the heart and kidney systems, providing a theoretical framework for applying TCM’s heart-kidney interaction theory to GII management.

From the above, this study is based on the theory of the heart and kidney and combined with previous research by our team, we selected HT7 (the original point of the hand-shaoyin heart meridian) and KI7 (the point of the foot-shaoyin meridian) as the main acupoints of interest in this study.

### 3.3 Methodological innovations

The Streitberger needle represents a specialized nonpenetrating placebo acupuncture device designed to simulate authentic acupuncture procedures without actual skin penetration. During application, patients typically perceive mild pressure or tapping sensations without genuine needle insertion, while the device maintains the therapeutic illusion through adhesive plastic rings securing it to the skin surface. This innovative tool provides credible sham control in clinical trials by effectively blinding participants to treatment allocation, as subjects cannot readily distinguish it from verum acupuncture. This design feature significantly minimizes patient-related bias and facilitates the differentiation between specific acupuncture effects and nonspecific placebo responses. Furthermore, the standardized application protocol ensures consistent implementation across studies, thereby reducing procedural variability in sham acupuncture interventions and enhancing methodological rigor in clinical research settings. The combination of the characteristics of effective participant blinding, an enhanced safety profile, and operational standardization establishes the Streitberger needle as a valuable methodological tool for investigating the neurobiological mechanisms underlying the therapeutic effects of acupuncture while controlling for placebo components in randomized controlled trials (36).

This methodological approach overcomes limitations identified in previous acupuncture insomnia research (67, 68), which frequently lacked both objective sleep measures and comprehensive assessment of comorbid symptoms. In alignment with international guidelines (69), our protocol incorporates objective ACT metrics, standardized psychometric instruments for mood and daytime function and biochemical markers (serum 5-HT) for mechanistic insights.

### 3.4 Study limitations

First, the single-center design and modest sample size limit generalizability to other settings and the precision of secondary and subgroup analyses. Second, requiring baseline PSG-derived TST < 6 h deliberately selects a short-sleep GII phenotype. Patients with clinically important insomnia characterized primarily by sleep fragmentation, prolonged wake after sleep onset, or low sleep efficiency despite TST ≥ 6 h will not be represented; therefore, the findings may not generalize to the broader GII population. Third, heterogeneity in glucocorticoid type, dose, duration, and timing is unavoidable. These variables will be documented, and the influence of major exposure changes will be evaluated in prespecified sensitivity analyses. Fourth, the sham device may produce tactile sensations that are not fully indistinguishable from verum needling and cannot eliminate all nonspecific effects. Fifth, although ISI, PSG-derived sleep efficiency, and PSG-derived TST are designated as key secondary endpoints and subjected to formal FDR control, all remaining secondary outcomes will be exploratory and interpreted cautiously. Peripheral serum 5-HT is also an exploratory biomarker and cannot be interpreted as a direct surrogate of central serotonergic activity. Finally, PSG will be performed only at baseline and immediately post-treatment, whereas the 1-week and 1-month follow-up assessments will rely on subjective questionnaires. This pragmatic design limits assessment of the persistence of objective changes in sleep architecture; even if subjective improvement is sustained, the durability of physiological effects will remain uncertain. The 1-month follow-up also precludes evaluation of longer-term durability.

In conclusion, this two-arm randomized controlled trial will evaluate the efficacy and safety of acupuncture for glucocorticoid-induced insomnia and will use a multidimensional assessment strategy to explore potential associated mechanisms. The findings are expected to inform the design of future confirmatory studies and clinical research in this population.

## 4 Trial status

The protocol was finalized on 1 March 2024, and ethics approval was granted by the ethics committee of the First Affiliated Hospital of Zhejiang Chinese Medical University on 24 April 2024 (no. 2024-KLS-089-02). The trial was registered with the International Traditional Medicine Clinical Trial Registry on 18 February 2025 (ITMCTR2025000220; pre-results), and recruitment began on 20 February 2025. Recruitment is ongoing, and study completion is anticipated by 20 December 2026.

## Glossary

ACT: Actigraphy
BAI: Beck Anxiety Inventory
BDI: Beck Depression Inventory
CBT-I: Cognitive behavioural therapy for insomnia
ESS: Epworth Sleepiness Scale
FAS: Full Analysis Set
FSS: Fatigue Severity Scale
GCs: Glucocorticoids
GII: Glucocorticoid-induced insomnia
HPA: Hypothalamic–pituitary–adrenal
ISI: Insomnia Severity Index
ITT: Intention-To-Treat
NREMS: Nonrapid eye movement sleep
PPS: Per Protocol Set
PSG: Polysomnography
PSQI: Pittsburgh Sleep Quality Index
REMS: Rapid eye movement sleep
RCT: Randomized controlled trial
SS: Safety Set
TCM: Traditional Chinese Medicine
TST: Total sleep time
TWT: Total wake time
5-HT: 5-hydroxytryptamine
